# Genital Infiltrations of CD4^+^ and CD8^+^ T Lymphocytes, IgA^+^ and IgG^+^ Plasma Cells and Intra-Mucosal Lymphoid Follicles Associate With Protection Against Genital *Chlamydia* *trachomatis* Infection in Minipigs Intramuscularly Immunized With UV-Inactivated Bacteria Adjuvanted With CAF01

**DOI:** 10.3389/fmicb.2019.00197

**Published:** 2019-02-08

**Authors:** Karin Erneholm, Emma Lorenzen, Sarah Bøje, Anja Weinreich Olsen, Gregers Jungersen, Henrik E. Jensen, Joseph P. Cassidy, Peter Andersen, Jørgen S. Agerholm, Frank Follmann

**Affiliations:** ^1^Section of Veterinary Reproduction and Obstetrics, Department of Veterinary Clinical Sciences, Faculty of Health and Medical Sciences, University of Copenhagen, Frederiksberg, Denmark; ^2^Department of Infectious Disease Immunology, Statens Serum Institut, Copenhagen, Denmark; ^3^Department of Health Technology, Technical University of Denmark, Lyngby, Denmark; ^4^Section of Experimental Animal Models, Department of Veterinary Disease Biology, Faculty of Health and Medical Sciences, University of Copenhagen, Frederiksberg, Denmark; ^5^Pathobiology Section, School of Veterinary Medicine, University College Dublin, Dublin, Ireland

**Keywords:** porcine, chlamydia, pathology, histology, vaccine

## Abstract

The development of a vaccine against genital chlamydia in women is advancing, and the evaluation of *in situ* immune responses following vaccination and challenge infections is crucial for development of a safe and protective vaccine. This study employs the sexually mature minipig model to characterize the genital *in situ* immune response to *Chlamydia trachomatis* infection in pigs previously immunized intramuscularly with UV-inactivated *C. trachomatis* serovar D (UV-SvD) adjuvanted/formulated with CAF01 adjuvant compared to a CAF01-alone control group. Pigs immunized with UV-SvD were significantly protected against vaginal challenge with *C. trachomatis* on day 3 post inoculation and showed significantly higher cervical infiltrations of approximately equal numbers of CD4^+^ and CD8^+^ T-cells, and IgG^+^ and IgA^+^ plasma cells compared to adjuvant-alone immunized controls. These immunological signatures correspond to findings in mice and are similar to those described in female chlamydia patients. This proves important potential for the pig model in elucidating immunological *in situ* signatures in future translational research in chlamydia vaccinology.

## Introduction

*Chlamydia* *trachomatis* is one of the most common sexually transmitted bacteria in the world, causing infertility due to development of chronic lesions in the upper genital tract (Haggerty et al., 2010). Research into its pathogenesis and to identify preventive strategies has been ongoing for many years, primarily in murine and guinea pig models (Rank and Whittum-Hudson, 1994; Maxion et al., 2004; O’Meara et al., 2014). However, while murine models in several aspects are very useful, mice have important differences in their immune system compared to humans making interpretation of immunogenicity and pathophysiology challenging (Meurens et al., 2012; De Clercq et al., 2013; Lorenzen et al., 2015b). Nonhuman primates (NHPs) are in general more comparable to humans than mice and have been used in immunogenicity studies with *C. trachomatis* serotypes. However, working with NHPs is associated with high costs and ethical restrictions (Bell et al., 2011; De Clercq et al., 2013). Pigs are increasingly being used for biomedical research, as their size, anatomy, physiology and immunology in many ways are comparable to those of humans (Fairbairn et al., 2011; Dawson, 2012; Meurens et al., 2012; Lorenzen et al., 2015b; Käser et al., 2017). Prepubertal pigs have previously been used to study genital chlamydial infection (Vanrompay et al., 2005; Schautteet et al., 2011b). However, the genital tracts of prepubertal and sexually mature pigs differ in size, epithelial thickness, vascularization, immune cell infiltration and hormone fluctuations (Dyck and Swierstra, 1983; Hickey et al., 2011; Lorenzen et al., 2015b, 2016) and the use of sexually mature pigs as models should replicate events occurring in women more closely.

Traditionally, vaccine immunogenicity and efficacy are evaluated by systemic/mucosal immune responses through e.g., flow cytometry and enzyme-linked immunosorbent assays and the level of microorganisms and/or absence of pathology, respectively. Histopathological evaluation, however, can reveal detailed information on how the local *in situ* immune responses are localized and if pathological changes are present. Such responses are particularly important for diseases of the genital tract, where an exacerbated response in the oviducts can cause infertility, whereas a strong local immune reaction in the cervix might be protective (Paavonen and Eggert-Kruse, 1999; Maldonado et al., 2014). Furthermore, the formation of memory lymphocyte clusters in the genital tract is implied to be crucial for chlamydia protection (Morrison and Morrison, 2000; Johnson and Brunham, 2016; Johnson et al., 2018), adding further arguments/motivation to perform immunohistochemical (IHC) analysis on genital tract tissues when evaluating potential chlamydia vaccines.

In this study, we investigate how immunization with whole UV-inactivated *C. trachomatis* serovar D bacteria (UV-SvD) adjuvanted with CAF01 compared to CAF01 alone, influences the genital *in situ* lymphocyte response to genital *C. trachomatis* infection. The detailed aim of the study was to evaluate and identify potential *in situ* immune cell signatures that may correlate with protection against chlamydial infection in the sexually mature minipig model of genital *C. trachomatis* infection.

## Materials and Methods

### *Chlamydia* *trachomatis*

*Chlamydia trachomatis* SvD (Trachoma type D strain UW-3/Cx, ATCC VR-885^TM^) was propagated in HeLa cells, harvested and purified essentially as previously described (Olsen et al., 2006). Inactivation of bacteria was achieved by exposing the bacterial solution to UV light at a distance of 5 cm for 3 h (Lu et al., 2002) and verification of the inactivation of infectivity was performed by inoculating the bacterial suspension onto McCoy cells and confirming the absence of inclusion forming units (IFU). The total protein content of the inactivated bacterial suspension was quantified by the bicinchoninic acid (BCA) method, as described in the Micro BCA^TM^ Protein Assay Kit (cat no 23235, Thermo Scientific) and used to adjust the concentration of the bacterial suspension for intramuscular immunization.

### Adjuvant and Vaccine Preparation

The adjuvant CAF01, a cationic liposome-based adjuvant, consisting of 500 μg glycolipid trehalose 6,6^′^-dibehenate (TDB) and 2500 μg dimethyldioctadecylammonium bromide (DDA), was prepared as described elsewhere (Hansen et al., 2008). UV-SvD bacteria were diluted in Tris–buffer (10 mM, pH 7.4) and mixed with the adjuvant (1:1), using sterile procedures.

### Experimental Animals

Sexually mature, 5–6 months-old female specific pathogen free Göttingen minipigs (Ellegaard Minipigs, Sorø, Denmark) were housed in the laboratory animal facilities at the Faculty of Health and Medical Sciences, University of Copenhagen, in groups of a maximum of five animals, were fed twice daily with standard minipig diet and had access to water *ad libitum*. Some of the pigs also served as control animals in another study (Bøje et al., 2015).

### Immunization, Infection Challenge and Sampling *in vivo*

The study was performed as two separate experiments due to logistics, with 4–5 pigs/group in each experiment. The pigs were intramuscularly immunized with 125 ug UV-SvD in CAF01 adjuvant (UV-SvD/CAF01) (*n* = 8) or with the adjuvant alone, (CAF01) (*n* = 9), as a control group, twice with an interval of 2–3 weeks (Table 1). Following the immunization the pigs were estrus synchronized with Regumate Equine^®^ (MSD Animal Health, Ballerup, Denmark) and 3–4 weeks after the last immunization, the animals were anesthetized during estrus with a Zoletil^®^ 50-mixture as described by (Lorenzen et al., 2015a), and inoculated deep intravaginally with 4 × 10^9^ IFU *C. trachomatis* SvD in 5 ml 250 mmol/l sucrose, 10 mmol/l NaH_2_PO_4_, and 5 mmol/l L-glutamic acid (SPG) using an insemination catheter (Osiris, E-vet, Denmark). The rear aspect of each inoculated pig was slightly elevated for 20 min post inoculation (pi) to reduce reflux of inoculum.

**Table 1:** Experimental design.

No of pigs	Immunization group	Timepoint			
		Week 0	Week 2–3	Week 5–7	
				Day 0 pi	Day 12 or 14 pi
9	CAF01	Immunization 1	Immunization 2	Vaginal inoculation	Euthanasia
8	125 μg UV-SvD/CAF01				

*Sexually mature female minipigs were immunized twice with either adjuvant alone (CAF01, *n* = 9) or UV-inactivated *C. trachomatis* SvD together with CAF01 (UV-SvD/CAF01, *n* = 8). Three to four weeks after the last immunization, the pigs were vaginally inoculated with 4 × 10^*9*^ IFU *C. trachomatis* SvD during estrus, and samples for histological analysis were obtained following euthanasia at day 12 or 14 pi*.

The pigs were clinically monitored and had their rectal temperature taken every second day in the post inoculation period: animals with body temperatures exceeding 39.5°C were considered pyrexic. Any changes in behavior, appetite, urination, defecation, vulval appearance or vaginal discharge were recorded.

Vaginal swabs were taken prior to inoculation and at euthanasia (week 2 pi) to monitor the level of mucosal antibodies and presence of *C. trachomatis* in the pi period. For antigen detection, additional vaginal swabs were taken on day 3 pi and week 1 pi as well. The swabs were immediately placed in 1 ml SPG and kept on ice until further handling. Three glass beads were added to each sample tube, and the samples were whirl mixed for 1 min, where after the swab material was aliquoted and stored at -80°C until further analysis. Blood samples were taken prior to inoculation and at 2 weeks pi, from the jugular vein in heparin-stabilized tubes for cell isolation and plain tubes for serum isolation. The plain tubes were centrifuged for 15 min at 2400 g, and serum was isolated and kept at -20°C until further handling.

### q-PCR Detection of *C. trachomatis*

The vaginal swabs for q-PCR detection were treated slightly differently in the two separate experiments, however, all samples from day 3 pi were treated similarly: DNA extraction was performed with Chelex^®^100 (Bio-Rad, Life Science, Denmark) and real-time q-PCR detection of *C. trachomatis* was performed by detection of the 16S rRNA gene as described in (Bøje et al., 2015; Erneholm et al., 2016). Based on the negative control, the C_t_ cut-off for true positive samples was determined to be 37.

### Measurement of *C. trachomatis* Specific Antibodies in Serum and in Vaginal Swabs

Determination of antigen-specific antibodies in serum and vaginal swabs was carried out using an indirect ELISA coating with UV-inactivated *C. trachomatis* SvD (4 μg/ml) as described previously (Erneholm et al., 2016). Confirmed positive serum from a previous study was included as a positive control and used as an internal standard to correct for plate-to-plate variation. Results are presented as corrected OD values, calculated as the increase in OD-value on day 14 pi compared to day 0 pi (corrected OD_day14pi_ = OD_day14pi_ – OD_day_ _0pi_).

### IFN-γ Response From *in vitro* Stimulated PBMCs and Lymph Node Cells

Peripheral blood mononuclear cells (PBMC) and lymph node cells were isolated and stimulated with UV-SvD (10 μg/ml), medium alone and staphylococcal enterotoxin B (1 μg/ml). After 3 days of incubation at 37°C with 5% CO_2_, cell culture supernatants were harvested and stored at -20°C until further analysis. The amounts of IFN-γ in cell culture supernatants were determined by a sandwich ELISA as described in (Bøje et al., 2015; Lorenzen et al., 2015a).

### Necropsy

At 12 or 14 days pi, the pigs were anesthetized and euthanized by exsanguination. At necropsy, all abdominal and thoracic organs were assessed for gross lesions. Samples from the vagina, cervix, uterine horns, and oviducts were fixed in 10% neutral buffered formalin and parallel samples were placed in cryostat embedding media (Tissue-Tek^®^ O.C.T., WVR) snap-frozen in ice cold petroleum, and stored at -80°C.

### Tissue Handling and Histology

The formalin fixed tissue was transferred to 70% ethanol after 48 h of fixation, processed routinely and embedded in paraffin. Sections of 3–4 μm were cut, mounted on SuperFrost^®^/Plus glass slides (Hounisen, Denmark), deparaffinized and rehydrated according to standard protocols and stained with hematoxylin and eosin (HE).

The histological slides were blinded, randomized and evaluated by the same observer. Infiltrations of lymphocytes, plasma cells and neutrophils in the vaginal, cervical and uterine mucosa were scored using a multiparametric semi-quantitative scoring system ranging from 0 to 4: 0: no/single cells, 1: scattered cells, 2: moderate numbers of cells, and/or few, small cell accumulations, 3: large numbers of cells, and/or multiple cell accumulations, 4: excessive numbers of cells, coalescing accumulations. Perivascular lymphocytic infiltrations were similarly scored from 0 to 3; 0: no infiltration, 1: mild infiltration/single vessel, 2: moderate infiltration/multiple vessels, 3: extensive infiltration, multiple vessels. Regarding the uterine horns, a mean value of each parameter for left and right horns was calculated. For statistical analysis, the scores for “lymphocytes” and “perivascular lymphocyte infiltration” were added for each sample. Presence of edema, degenerated epithelium, and luminal content were also recorded.

The frozen tissues were cut on a cryostat, fixed in ice cold acetone for 20 min and air dried. The sections were kept at -20°C until further handling.

### Histochemical and Immunohistochemical Staining

Representative histosections were selected for visualization of plasma cells, T cells and T cell subsets. For visualization of plasma cells, the slides were incubated in methyl green pyronin solution (MGP) (Sigma-Aldrich) for 10 min, rinsed in distilled water, and air dried and mounted in xylene.

For visualization of T cells (CD3^+^), helper T cells (CD4^+^), and cytolytic T cells (CD8^+^), as well as isotype-specific plasma cells through membrane-bound IgA and IgG, immunohistochemistry was performed, see Table 2 for details. The anti-CD8 antibody used binds the b epitope of the CD8α chain. In pigs, CD8α is constitutively expressed on cytolytic T cells and expressed upon antigen activation of CD4 and γδ T cells (Gerner et al., 2009). Blocking and incubation steps were performed according to the manufacturers’ recommendations. A negative control was included using a non-sense antibody (goat serum for IgA and IgG, isotype specific IgG for CD3 (mouse IgG_1_, Clone P3.6.2.8.1, eBioscience), CD4 (mouse IgG_2b_, clone MPC-11, BD Pharmingen) and CD8 (mouse IgG_2a_, clone C1.18.4, BD Pharmingen). In addition, positive control sections were included. The complete list of antibodies used for IHC is included in Table 2.

**Table 2:** Overview of immunohistochemical antibodies and reagents.

Target antigen	Manufacturer	Dilution and incubation of primary antibody	Detection method and chromogen
**Formalin fixed tissue**			
Mouse anti-pig CD3	Southern Biotech Clone PPT3	1:1000 o/n 4 °C	Ultravision One DAB^a^
Goat anti-pig IgA	Nordic Biosite Lot no: A100-102A-16	1:4000 1 h RT	Vectastain Elite-ABC – Goat AEC^b^
Goat anti-pig IgG	Nordic Biosite Lot no: A100-104A-12	1: 7000 1 h RT	Vectastain Elite-ABC – Goat AEC^b^
**Frozen tissue**			
Mouse anti-pig CD4	Southern Biotech Clone 74-12-4	1:500 1 h RT	Ultravision AEC
Mouse anti-pig CD8α	BD Pharmingen Clone 295/33-25	1:500 1 h RT	Ultravision AEC

*All formalin fixed tissue was exposed to antigen retrieval by microwave boiling in ^*a*^TEG buffer pH 9.0 for 2 × 5 min, ^*b*^Citrate buffer pH 6.0 for 5 min, and blocked for endogenous peroxidase in a solution of hydrogen peroxide (0.6%) for 15 min. RT = room temperature*.

### Statistics

Statistical analyses were carried out using Graph Pad Prism 5. The Mann-Whitney test was used to analyze differences between the groups. *P* < 0.05 was considered statistically significant. The following symbols were used to designate significance: ^∗^*P* 0.01 to 0.05, ^∗∗^*P* 0.01–0.001, ^∗∗∗^*P* < 0.001.

### Ethics Statement

This study was carried out in accordance with the recommendations of the EU Directive 2010/63, Danish Animal Experiments Inspectorate of the Danish Veterinary and Food Administration. The protocol was approved by the Danish Animal Experiments Inspectorate with license no: 2008/561-1581.

## Results

### *Chlamydia trachomatis* Infectious Load Following Inoculation

**FIGURE 1:**
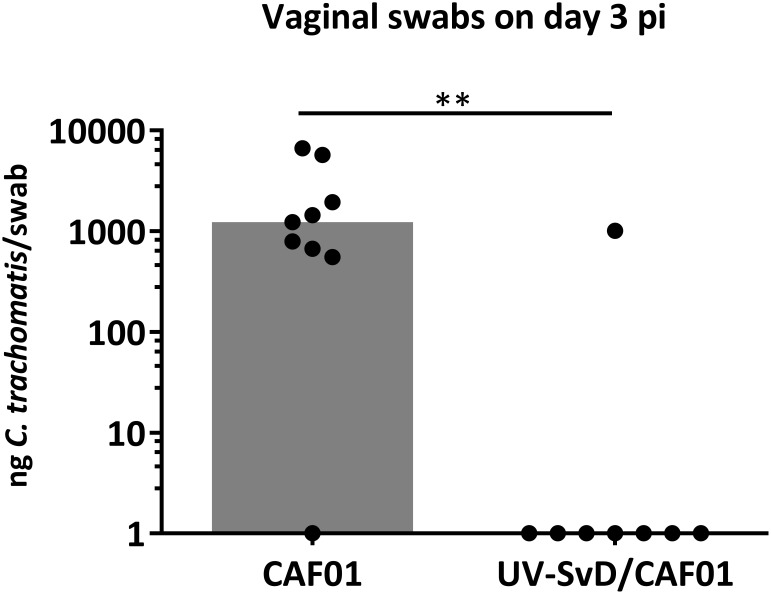
*Chlamydia trachomatis* q-PCR detection in vaginal swabs. Detection of *C. trachomatis* (*Ct*) in vaginal swabs by q-PCR on day 3 pi. Each dot represents one animal. The gray bar shows median. Statistics: Mann-Whitney test. Two asterisks (^∗∗^) indicate a *P* value between 0.01–0.001.

Pigs were intramuscularly immunized with 125 ug UV-SvD in CAF01 (UV-SvD/CAF01) (*n* = 8) and the control group was administered adjuvant alone (CAF01) (*n* = 9) by the intramuscular route. The animals were challenged during estrus by deep vaginal inoculation with 4 × 10^9^ IFU *C. trachomatis* SvD and all pigs remained clinically unaffected throughout the study. Following vaginal challenge, infectious load was determined in vaginal swabs by q-PCR, detecting *C. trachomatis* 16S rRNA genes. The immunization with UV-SvD/CAF01 induced protection in terms of a significantly lower vaginal bacterial load on day 3 pi in the UV-SvD/CAF01 group, compared to the control/CAF01 group (Figure 1). On the next sampling day (i.e., day 7 pi), only 2 out of 9 animals in the CAF01 group and 1 out of 8 animals in the UV-SvD/CAF01 group had detectable levels of vaginal *C. trachomatis* by q-PCR. On the following sampling (days 12 or 14 pi) 1 out of 9 animals in the CAF01 group and 2 animals in the UV-SvD/CAF01 group had detectable levels of vaginal *C. trachomatis* (Supplementary 1).

### Necropsy and Histopathological Examination of the Genital Tract

**FIGURE 2:**
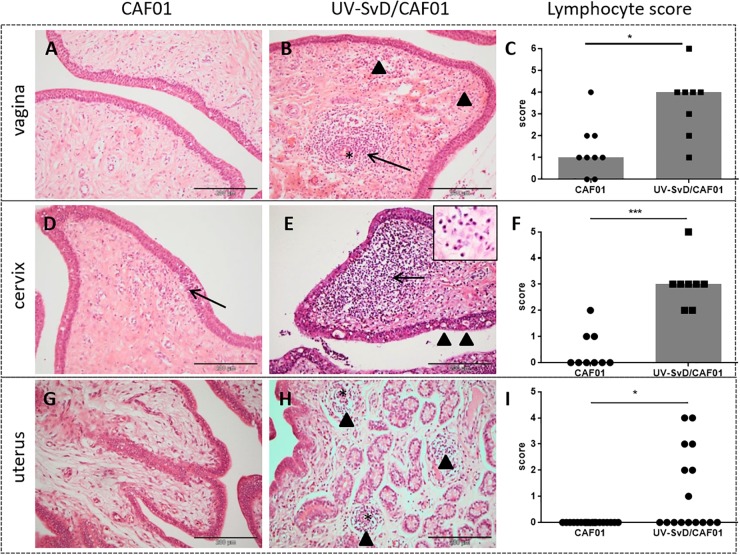
Histopathological changes including lymphocyte counts in the minipig vagina, cervix and uterus. The first and second column show representative pictures from the CAF01 and the UV-SvD/CAF01 groups, respectively. Each row represents the different anatomical locations; the vagina, cervix and uterus. The scale bar equals 200 μm. **(A)** In the vagina of the CAF01-group, there is a low number of lymphocytes scattered in the subepithelial stroma. **(B)** UV-SvD/CAF01 group, vagina. A profound perivascular infiltration of lymphocytes is noted (arrow), and also a subepithelial diffuse infiltration of lymphocytes (arrowheads). Blood vessel marked with asterisk (^∗^) **(C)** Comparison of the semiquantitative lymphocyte infiltration scores, showing a significant difference in the lymphocyte score in the vagina of the two groups. Each dot represents one animal. Bars show median. **(D)** CAF01-group, cervical tissue, where a small subepithelial lymphocyte infiltration is extending into the epithelium (arrow) **(E)** In the cervix of the UV-SvD/CAF01 group, there is a dense subepithelial infiltration of lymphocytes (arrow) and plasma cells (insert, arrow). Intraepithelial vacuoles (arrowheads) and lymphocytes are also noted **(F)** Comparison of the semiquantitative lymphocyte infiltration scores, showing a highly significant difference in the lymphocyte score in the cervix of the two groups. Each dot represents one animal. Bars show median. **(G)** In the uterus, stromal edema is found in the CAF01-group **(H)** In addition to stromal edema, multiple small perivascular lymphocytic accumulations (arrowheads) are seen in the UV-SvD/CAF01 group. Blood vessel marked with asterisk (^∗^) **(I)** Comparison of the semiquantitative lymphocyte infiltration scores, showing a significant difference in the lymphocyte score in the uterus of the two groups. Each dot represents one uterine horn. Bars show median. Statistics: Mann-Whitney test. Asterisks (^∗^) indicate the level of significance: ^∗^*P* 0.01 to 0.05, ^∗∗^*P* 0.01–0.001, and ^∗∗∗^*P* < 0.001.

At necropsy, congestion was observed in the broad ligament of the uterus in pigs of both groups. In all animals, the uterine mucosa was edematous and slightly congested, and the ileosacral lymphocentrum was slightly enlarged. Pigs in the UV-SvD/CAF01 group had significantly greater infiltration of lymphocytes in the mucosa of the uterus (*p* = 0.0021), cervix (*p* = 0.0002), and vagina (*p* = 0.01) relative to the CAF01 group (Figure 2C,F,I). The lymphocytes were typically observed in moderate numbers, localized subepithelially, either scattered in the stroma, or aggregated in follicles (Figure 2). In the UV-SvD/CAF01 group, the lymphocytes were also located perivascularly in the mucosa (Figure 2B, arrow). Intraepithelial lymphocytes were found to a lesser extent in both groups (Figure 2E, arrowheads). A significantly denser infiltration of plasma cells was present in the cervix of the UV-SvD/CAF01 group, compared to the control group (Figure 2E insert, and Supplementary 2). The plasma cells were typically located in subepithelial clusters, intermingled with lymphocytes, and diffusely scattered in the subepithelial stroma. Neutrophils were noted in low numbers in the epithelium and subepithelial stroma of the vagina and cervix, in both groups (Figure 2A–E). Mild to moderate hydropic degeneration and cytoplasmic vacuolation, was a feature of the vaginal and cervical epithelium of both groups (Figure 2E, arrowheads), with accompanying mild to moderate intraepithelial microabcessation. The severity of epithelial degeneration was slightly greater in the cervix than in the vagina in both groups; however, no differences were found between the two groups. Lesions were not found in the oviducts of either groups.

### Characterization of Leukocyte Infiltration

**FIGURE 3:**
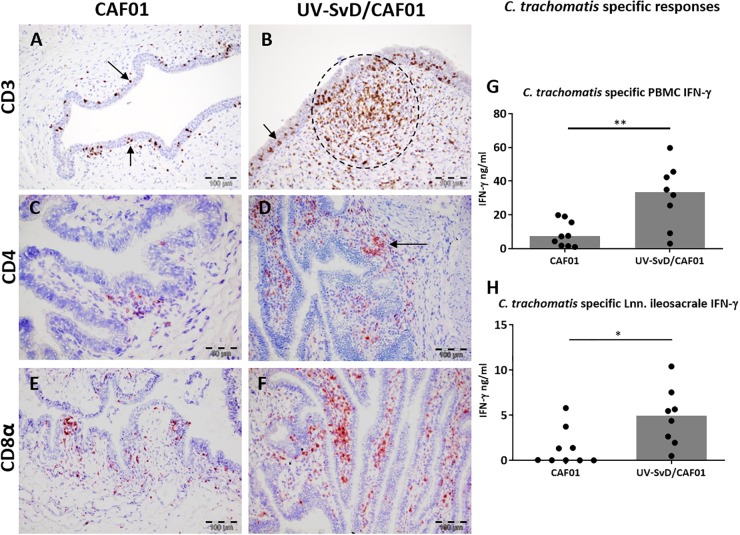
CD3^+^, CD4^+^, and CD8^+^ T cells in the cervix along with *C. trachomatis* specific PBMC IFN-*γ* responses. Representative sections from the cervix were immunohistochemically (IHC) stained against CD3, CD4, and CD8α. All pictures and graphs are from day 14 pi. The left column shows the adjuvant (CAF01) control group IHC stains, the middle column shows the UV-SvD/CAF01 IHC stains and the right column presents *C. trachomatis* specific immune responses in PBMCs **(G)** and lymph nodes **(H). CD3 (A,B)**: CD3^+^ cells (brown) were detected in all sections. **(A)** In the CAF01-group the CD3^+^ cells were mainly located intraepithelially (arrows). **(B)** In the UV-SvD/CAF01 group, the CD3^+^ cells were typically accumulated in the subepithelial stroma (encircled), and occasionally also intraepithelially (arrow). **CD4 (C,D):** CD4^+^ cells (staining red) were found in both groups, localized in subepithelial lymphoid accumulations (**D**, arrow), and occasionally in the epithelium; however, to a larger degree in the UV-SvD/CAF01 group **(D)**. **CD8 (E,F)**: CD8^+^ cells were found both intraepithelially and subepithelially in both groups (arrows), with a larger amount in the UV-SvD/CAF01 group, in a similar fashion to the CD4^+^ cells. In lymphoid accumulations, primarily present in the UV-SvD/CAF01 group, the density of CD4^+^ and CD8^+^ cells were approximate equal (also illustrated in supplementary 3). The right column shows *C. trachomatis* specific immune responses. Each dot represents one animal. The gray bar shows the median **(G)** Systemic T-cell response: IFN-γ response from PBMCs re-stimulated with *C. trachomatis* **(H)** T-cell response in the uterine draining lymph node: IFN-γ response from lymph node cells re-stimulated with *C. trachomatis* Statistics: Mann-Whitney test. Asterisks (^∗^) indicate the level of significance: ^∗^*P* 0.01 to 0.05, ^∗∗^*P* 0.01–0.001, and ^∗∗∗^*P* < 0.001.

To further analyze the leukocyte infiltration in the lower genital tract, representative sections were selected and stained immunohistochemically with anti-CD3 for detection of T cells, and with MGP to confirm the presence of plasma cells. As visualized in Figure 3, the cell accumulations in the stroma were to a large extent made up of CD3^+^ cells, particularly in the UV-SvD/CAF01 group, where these cells were most dense (Figure 3B). CD3^+^ lymphocytes were also found intraepithelially in the cervix and vagina of both groups (Figure 3A,B, arrowheads). While plasma cells were present in lower numbers compared to CD3^+^ cells, they were more of a prominent feature in the UV-SvD/CAF01 group than in the CAF01-group (Supplementary 2). Plasma cells were typically found in aggregates and in more dispersed scattering in the sub-epithelial stroma. For further characterization of T cells and plasma cells, IHC staining of CD4, CD8, IgA, and IgG were performed in selected, representative, sections of the vagina, cervix and uterus of both groups. In general, there were more CD8^+^ T cells than CD4^+^ T helper lymphocytes in both groups (Figure 3C–F). The overall distribution of CD4^+^ and CD8^+^ cells overlapped consistently with the CD3 staining, with the highest number of cells found in the vagina and cervix of UV-SvD/CAF01 immunized pigs (Figure 3A–F). These cells predominantly appeared as accumulations in the subepithelial stroma (Figure 3). The density of CD4^+^ and CD8^+^ cells was similar in these accumulations (Supplementary 3). Whereas the CD4^+^ cells were almost only observed in the accumulations, CD8^+^ cells also localized intraepithelially, with a multifocal distribution, corresponding to the intraepithelial staining of CD3 (Figure 3C–F).

**FIGURE 4:**
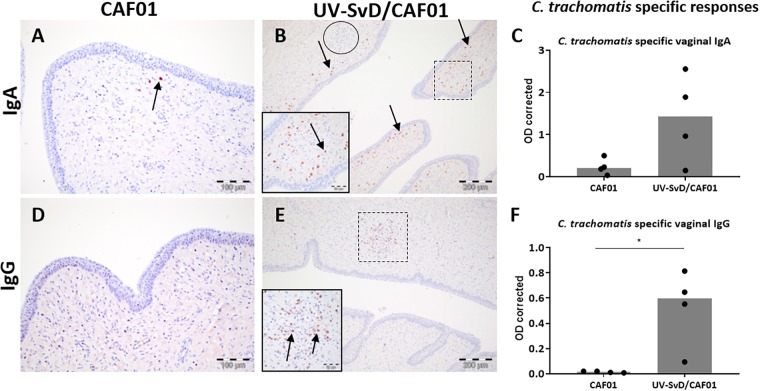
IgG^+^ and IgA^+^ B cells in the cervix along with vaginal *C. trachomatis* specific IgA and IgG antibody responses. Representative sections from the cervix were immunohistochemically (IHC) stained against IgA and IgG. All pictures and graphs are from day 14 pi. The left column shows the adjuvant (CAF01) control group IHC stains, the middle column shows the UV-SvD/CAF01 IHC stains and the right column presents *C. trachomatis* specific immune responses in vaginal swabs **(C,F)**. The UV-SvD/CAF01 group **(B,E)** contained more plasma cells than the CAF01 group **(A,D)** (arrows). In general, the IgA^+^ cells were more abundant than IgG^+^ cells. IgA^+^ cells were typically scattered in the subepithelial stroma (**B**, insert and arrows), whereas the lymphoid follicles had no, or very few IgA^+^ cells (**B**, lymphoid follicle encircled). IgG^+^ cells were typically found exclusively inside, or adjacent to, cellular accumulations (**E**, lymphoid follicle marked and inserted). The right column shows *C. trachomatis* specific immune responses. Each dot represents one animal. The gray bar shows the median **(C)** *C. trachomatis* specific IgA in vaginal swabs. Representative data from one experiment. **(F)** *C. trachomatis* specific IgG in vaginal swabs. Representative data from one experiment. Asterisks (^∗^) indicate the level of significance: ^∗^*P* 0.01 to 0.05, ^∗∗^*P* 0.01–0.001, and ^∗∗∗^*P* < 0.001.

The distribution of plasma cells was described in HE and MGP stained sections, with moderate numbers of this cell type infiltrating the vagina of both groups. A higher density of plasma cells was detected in the cervix of the UV-SvD/CAF01 group compared to the CAF01 group (Figure 4A–E). There were more IgA^+^ cells relative to IgG^+^ cells in the vagina and cervix of both groups. In the UV-SvD/CAF01 group, the IgA^+^ cells were scattered multifocally in the subepithelial stroma (Figure 4B). IgG^+^ cells were almost exclusively found within, or adjacent to, lymphoid follicles (Figure 4E). Few IgA^+^ and IgG^+^ cells were found in the uterine horns.

### *Chlamydia trachomatis* Specific Immune Responses Following Inoculation

The UV-SvD/CAF01 immunized pigs had significantly higher *Chlamydia*-specific IgG levels in serum on the day of infection (0 pi) (*p* = 0.0138) and on day 14 pi (*p* = 0.0291), compared to the CAF01 group (Supplementary 4). Furthermore, the UV-SvD/CAF01 immunized pigs had significantly higher *Chlamydia*-specific IgG levels on the vaginal surface on day 14 pi compared to the CAF01 control group (*p* = 0.0286, Figure 4F) and similarly these animals exhibited a significantly higher systemic IFN-γ response from re-stimulated PBMCs on day 14 pi (Figure 3G, *p* = 0.0079). In addition, the UV-SvD/CAF01 group also showed significantly higher IFN-γ responses in cells from the re-stimulated iliosacral lymph node at day 14 pi (Figure 3H, *p* = 0.015).

## Discussion

The essential aim of this study was to evaluate and identify potential *in situ* immune cell signatures that may correlate with protection against chlamydial infection in the sexually mature minipig model of genital *C. trachomatis* infection.

The genital *in situ* immune cell signature in the UV-SvD/CAF01 vaccinated group, associated with the significant protection on day 3 pi in this group, consisted of significant infiltrations of plasma cells and T cells in the sub-epithelial stroma of the lower genital tract, particularly in the cervical stroma. The immune cells were present in a scattered distribution as well as in sub-epithelial lymphoid follicles consisting of approximate equal numbers of CD4^+^ and CD8^+^ T cells, along with IgG^+^ and IgA^+^ plasma cells. While it is not a given that these immune cells are *C. trachomatis*-specific, the IHC results correlate well with the significant *C. trachomatis-*specific IFN-γ, IgG and IgA responses (Figure 3, 4) suggesting that the IHC data represent an *in situ* *C. trachomatis*-specific response.

The increased numbers of CD8^+^ T cells in the genital tract is consistent with previously described resident CD8^+^ cells in the porcine uterine epithelium (Kaeoket et al., 2001, 2002), which are analogous to intraepithelial T cells in human cervical epithelium (Johansson et al., 1999). Likewise, a larger proportion of CD8^+^ cells have been found in infected NHPs (Van Voorhis et al., 1996) and guinea pigs (Rank et al., 2000), whereas in mice, CD4^+^ T cells have been found to dominate following vaccination and *Chlamydia* challenge (Morrison and Morrison, 2000; Johansson and Lycke, 2001). Importantly, the composition of the cellular infiltrate in the pig model is similar to that reported in human cases of chlamydial cervicitis, including subepithelial inflammation with increased numbers of both CD4^+^ and CD8^+^ T cells, plasma cells and lymphoid follicle formation (Kiviat et al., 1990; Mittal et al., 2004; Ficarra et al., 2008; Agrawal et al., 2009). Hence, immunized pigs may mount an adaptive immune response *in situ* mimicking reinfection in human patients with chlamydial cervicitis. It should be noted that the anti-CD8 antibody used does not differentiate CD8^+^ single positive cytolytic T cells and antigen-activated CD8^+^/CD4^+^ double positive helper T cells. In pigs 10–13% of the T cells can be CD8^+^/CD4^+^ double positive, hence a proportion of the identified CD8^+^ cells might be double positive (Gerner et al., 2009, 2015). However, when comparing the distribution of CD4^+^ and CD8^+^ positive cells (Supplementary 3) it is clear that the intraepithelial T cells are predominantly CD8^+^ single positive cytolytic T cells.

The finding of IgA^+^ and IgG^+^ plasma cells in the genital tract following vaginal challenge upon intramuscular immunizations is promising from the perspective of an intramuscular immunization strategy, but contradicts to some extent recent studies indicating that a mucosal/intranasal booster immunization is needed to trigger significant IgA (Lorenzen et al., 2015a; Christensen et al., 2017) and T cell responses on the genital mucosa (Stary et al., 2015). However, other studies in pigs have also shown protection without the addition of a mucosal booster (Schautteet et al., 2011a; Bøje et al., 2015). Clearly, the route of administration influences the immune response (Rank et al., 1990; Lu et al., 2002; Stary et al., 2015) and for both mucosal as well as parenteral immunizations it is important to use the optimal adjuvant/delivery system to direct the appropriate immune response (Igietseme et al., 2011; Stary et al., 2015). In a comparative study of five clinically tested adjuvants for parenteral use (IC31^®^, CAF01, GLA-SE, MF59^®^ and Alum), Knudsen et al., 2016 demonstrated that CAF01 has an immunological profile (Th1/Th17) tailored for *Chlamydia.* Similarly, parenteral immunization with UV-EBs formulated in the (Th1) AS01B adjuvant can elicit significant protection (Coler et al., 2009). Future studies are required to elucidate the effect of immunization routes/adjuvants and the relative impact of effector T cells, genital IgA producing plasma cells and vaginal IgG on bacterial clearance. In such studies, the immune cell subsets could also be quantified.

An important perspective of evaluating *in situ* responses and cell signatures is to be able to distinguish between protective and aberrant cell mediated responses that cause oviduct pathology (Paavonen and Eggert-Kruse, 1999; Maldonado et al., 2014). In general, the vagina and especially the cervix function as immune-inductive sites, with formation of mucosa-associated lymphoid aggregates in response to local antigenic stimulation (Pudney et al., 2005). This is normally a non-pathologic response, protecting the genital tract from ascending infections (Pudney et al., 2005). Tissue-resident T cells are crucial for protective immunity in the genital tract, both against *Chlamydia* (Igietseme and Rank, 1991; Johansson and Lycke, 2001; Olive et al., 2011), and herpes simplex virus 2 (Iijima and Iwasaki, 2014). In fact it has recently been hypothesized that the establishment of *C. trachomatis*-specific memory lymphocyte clusters in the genital mucosa is crucial for protection against chlamydia infection (Stary et al., 2015; Johnson and Brunham, 2016). Furthermore, the development of tertiary lymphoid tissue at sites of inflammation has also been associated with increased protection at other anatomical locations exposed to microorganisms from the external environment (Wiley et al., 2009).

In the current study, few histopathological changes were observed in the uterine horns, and no lesions were found in the oviducts. It is likely that the bacteria did not ascend to the upper genital tract during this study, due to the short duration of infection and long and complex nature of the porcine cervix as discussed in (Lorenzen et al., 2015b). For true evaluation of upper genital tract cell signatures and development of pathology, the model should be optimized e.g., by inoculating directly into the uterus as recently performed (Lorenzen et al., 2017). Nonetheless, the observed inflammation in the porcine cervix is similar to what occurs in human chlamydial cervicitis, representing most of the human infections (Brunham and Rey-Ladino, 2005). Only a small percentage of asymptomatic cervical chlamydia infections are actually estimated to ascend the genital tract (Haggerty et al., 2010).

The systemic immune response was also evaluated in the present study, and the robust *Chlamydia*-specific antibody and IFN-γ responses in the UV-SvD/CAF01 vaccinated animals were consistent with previous studies with CAF01 (Agger et al., 2008) and are also similar to responses to immunization with UV-inactivated *Chlamydia* in guinea pigs (Rank et al., 1990) and mice (Lu et al., 2002).

## Conclusion

In conclusion, the significant protection of minipigs immunized with UV-SvD/CAF01 was associated with significant infiltrations of CD4^+^ and CD8^+^ T lymphocytes and IgA^+^ and IgG^+^ plasma cells to the lower genital tract with the attendant formation of intra-mucosal lymphoid follicles. These findings correspond to the “immune cell signatures” that develop during chlamydial cervicitis in women and highlight the potential use of this experimental infection model for more detailed evaluation of *in situ* responses during vaccine trials including its use in distinguishing pathologic from protective responses.

## Author Contributions

KE participated in the design of the study, the experimental work, data analyses, and draft of the manuscript. EL participated in the data analyses and draft of the manuscript. SB participated in the design of the study, the experimental work, data analyses, and critically revision of the manuscript. AO, GJ, HJ, JC, and PA were involved in the conception of the study, interpretation of data, and critically revision of the manuscript. JA and FF participated in the design of the study, interpretation of data, and the draft of the manuscript. All authors read and approved the final manuscript.

## Conflict of Interest Statement

PA is co-inventor on an issued patent (US8277823) covering the use of CAF01 as vaccine adjuvant. All rights have been assigned to Statens Serum Institut, a Danish not-for-profit governmental institute. The remaining authors declare that the research was conducted in the absence of any commercial or financial relationships that could be construed as a potential conflict of interest. The handling Editor declared a past supervisory role with the author KE.
