# Supplementary material for: Genital Infiltrations of CD4+ and CD8+ T Lymphocytes, IgA+ and IgG+ Plasma Cells and Intra-Mucosal Lymphoid Follicles Associate With Protection Against Genital Chlamydia trachomatis Infection in Minipigs Intramuscularly Immunized With UV-Inactivated Bacteria Adjuvanted With CAF01
Source: Front Microbiol. 2019 Feb 8;10:197. doi: 10.3389/fmicb.2019.00197 (PMC6375829; doi:10.3389/fmicb.2019.00197)
Supplement: Supplementary file 1 [file Data_Sheet_1.pdf]

Supplementary 1. Number of animals with detectable levels of vaginal *C. trachomatis* post infection

	d. 7 pi	d. 12 or 14 pi
CAF01 (n=9)	2/9	1/9
UV-SvD/CAF01 (n=8)	1/8	2/8

Detection of *C. trachomatis* in vaginal swabs by q-PCR on day 7 pi and either day 12 or 14 pi. The numbers indicate the number of animals with detectable levels of *C. trachomatis* out of the total number of animals. Only very few animals (1 or 2) had detectable levels of *C. trachomatis* on day 7 pi and 12 or 14 pi.

Supplementary 2. Semiquantitative scoring of plasma cell infiltrates in the cervix

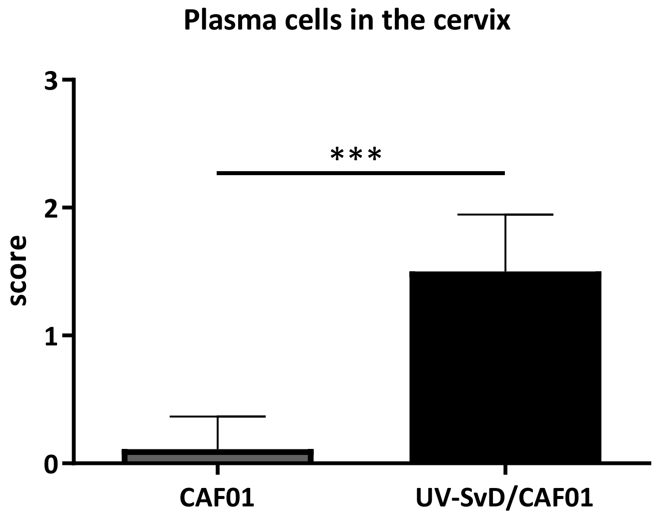

Semiquantitative scoring of cellular infiltrates. The cellular infiltrates were given a severity score from 0 (none/single cells) to 4 (excessive numbers). Bars show mean values of the group, error bars 95% CI. Statistics: Mann Whitney

Supplementary 3. Comparison of CD4 and CD8 IHC on parallel sections

CD4 CD8

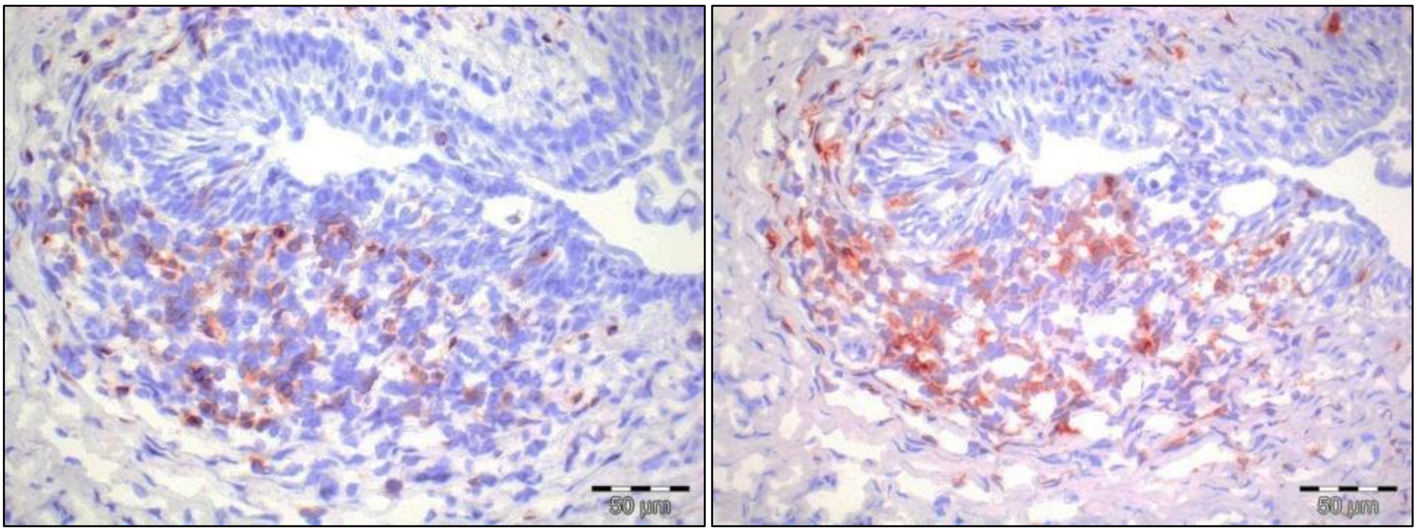

Immunohistochemical staining of CD4 and CD8 T cells on parallel sections from the vagina of a pig from the UV-SvD/CAF01 group. It shows similar densities of each cell type within subepithelial lymphoid accumulations. The intraepithelial T cells are almost exclusively $CD8^{+}$.

Supplementary 4. *C. trachomatis* specific serum IgG response

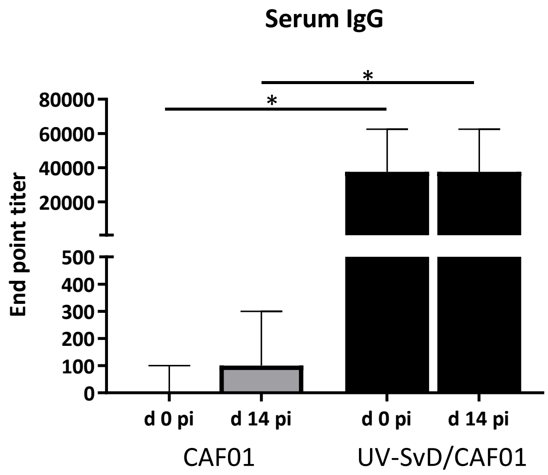

Representative data on *C. trachomatis*-specific serum IgG. The UV-SvD/CAF01 vaccinated group showed significant higher levels of *C. trachomatis* specific serum IgG at day 0 and at day 14 pi. Bars show mean and SD.
